# Supplementary material for: A Whole-Genome Analysis of the African Swine Fever Virus That Circulated during the First Outbreak in Vietnam in 2019 and Subsequently in 2022
Source: Viruses. 2023 Sep 18;15(9):1945. doi: 10.3390/v15091945 (PMC10537361; doi:10.3390/v15091945)

Figure S1. Sequence alignment of open reading frames(ORFs) compared between VN/HY/2019-ASFV1(represented by VN_HA-ASFV1) and VN/QP/2019-ASFV1(represented by VN_QP-ASFV1 with other ASFV strains.

(a) Sequence Alignment of EP153R nucleotides

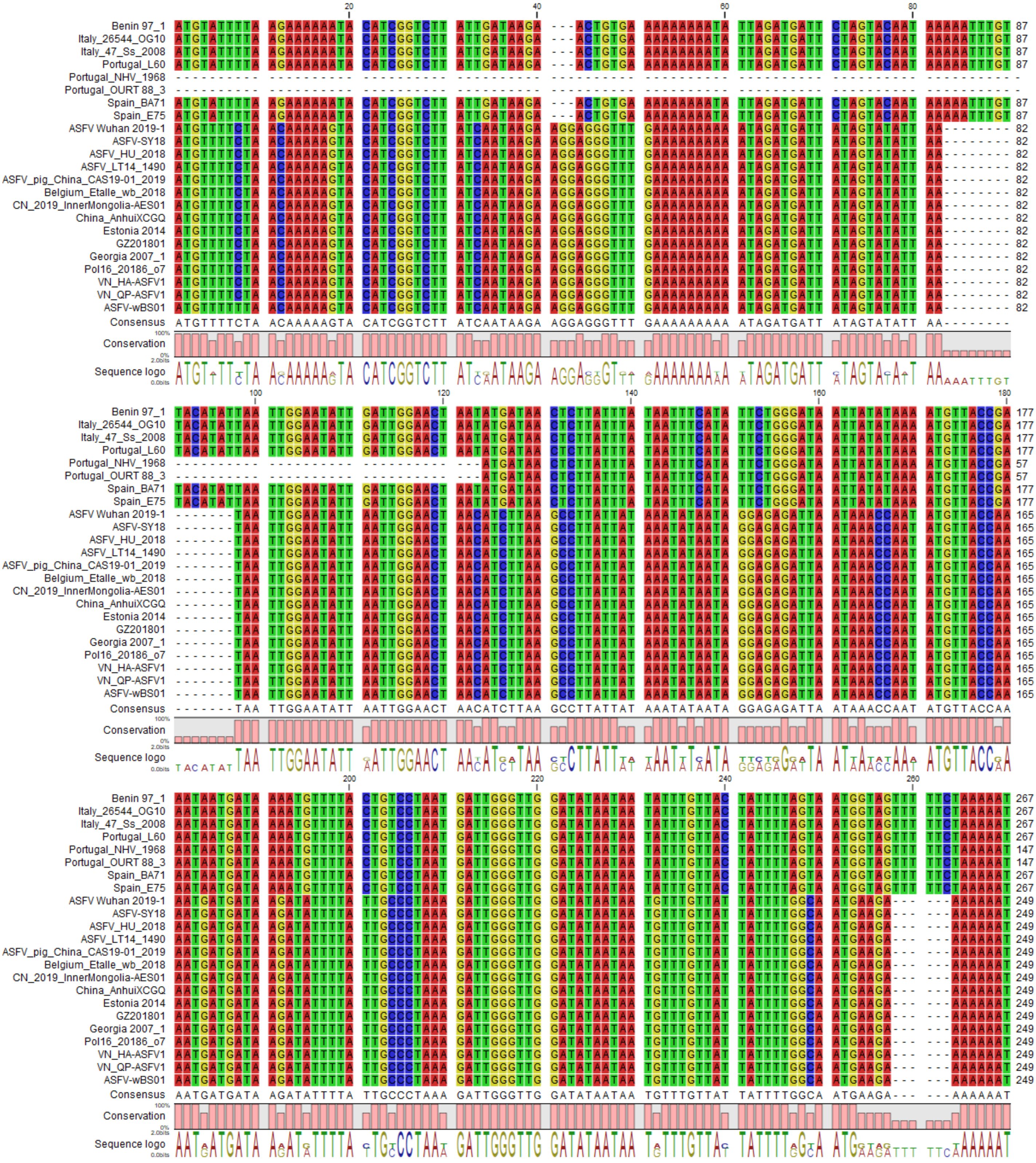

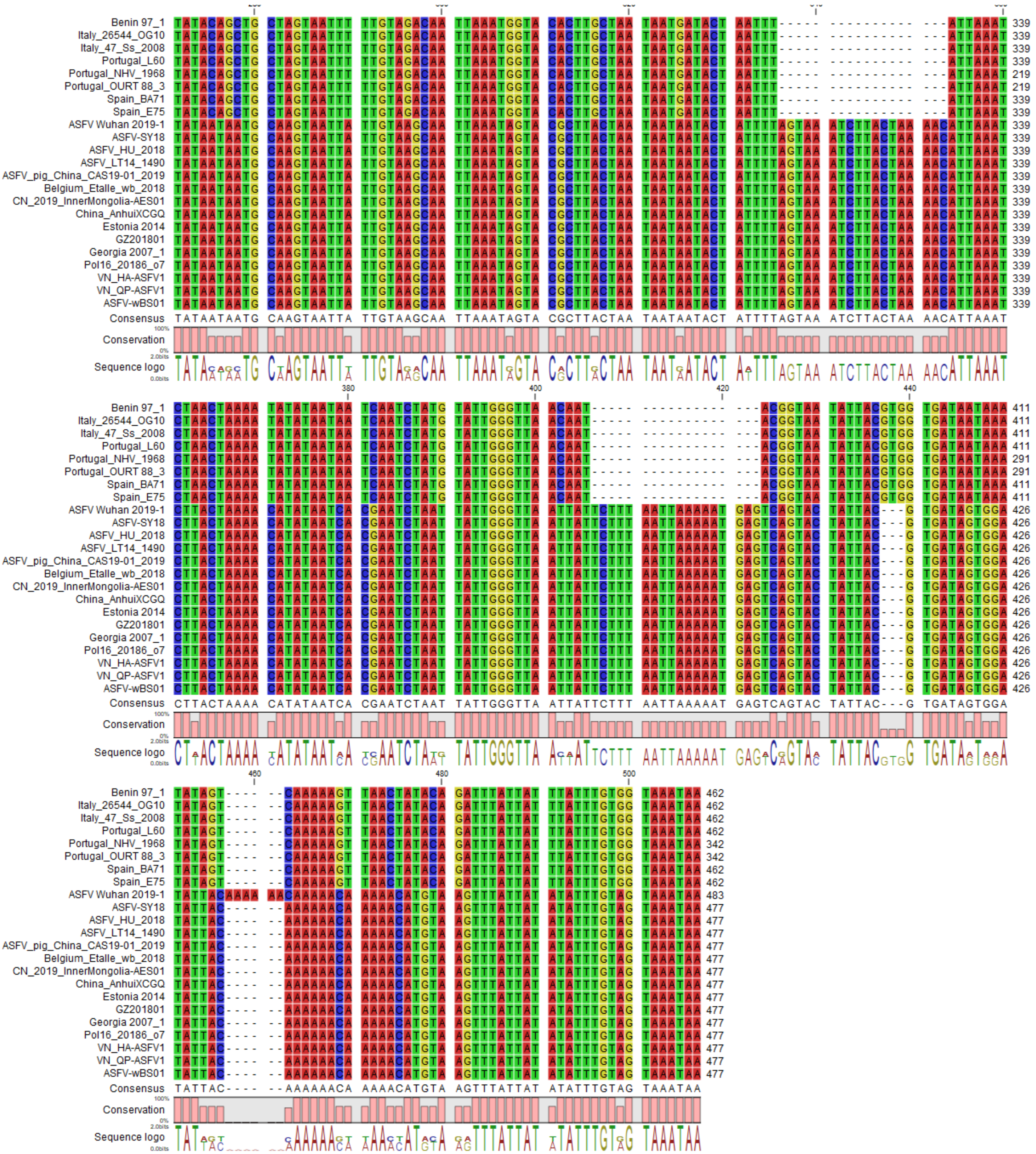
Benin 97_1
Italy_26544_OG10
Italy_47_Ss_2008
Portugal_L60
Portugal_NHV_1968
Portugal_OURT 88_3
Spain_BA71
Spain_E75
ASFV Wuhan 2019-1
ASFV-SY18
ASFV_HU_2018
ASFV_LT14_1490
ASFV_pig_China_CAS19-01_2019
Belgium_Etalle_wb_2018
CN_2019_InnerMongolia-AES01
China_AnhuiXCGQ
Estonia 2014
GZ201801
Georgia 2007_1
Pol16_20186_o7
VN_HA-ASFV1
VN_QP-ASFV1
ASFV-wBS01
Consensus TATAATAATG CAAGTAATTA TTGTAAGCAA TTAAATAGTA CGCTTACTAA TAATAATACT ATTTTAGTAA ATCTTACTAA AACATTAAAT
Conservation
Sequence logo
380
400
420
440
Consensus CTTACTAAAA CATATAATCA CGAATCTAAT TATTGGGTTA ATTATTCTTT AATTAAAAAT GAGTCAGTAC TATTAC---G TGATAGTGGA
460
480
500
Consensus TATTAC---- --AAAAAACA AAAACATGTA AGTTTATTAT ATATTTGTAG TAAATAA

(b) Sequence Alignment of EP153R proteins

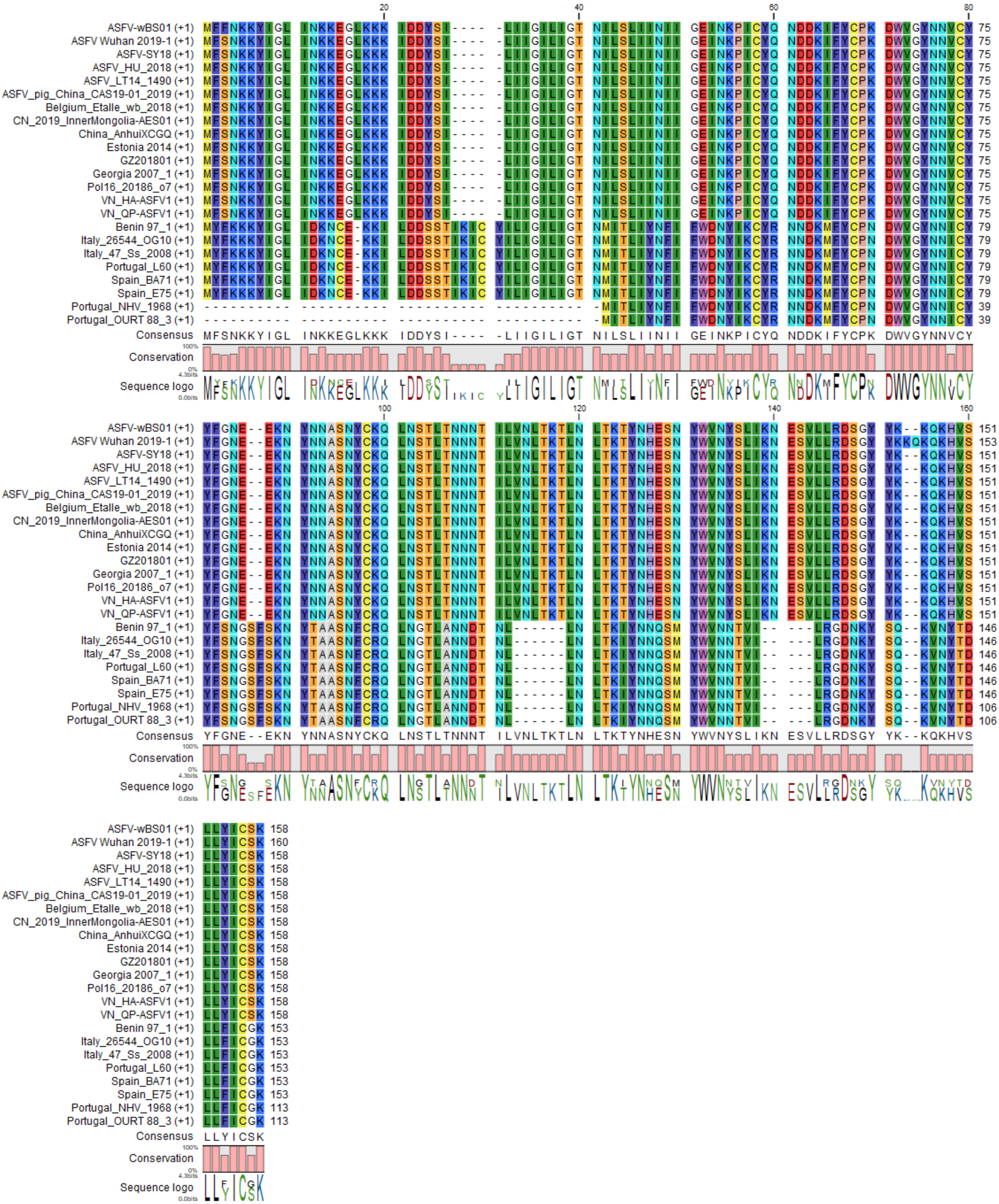

(c) Sequence Alignment of MGF360-1L nucleotides

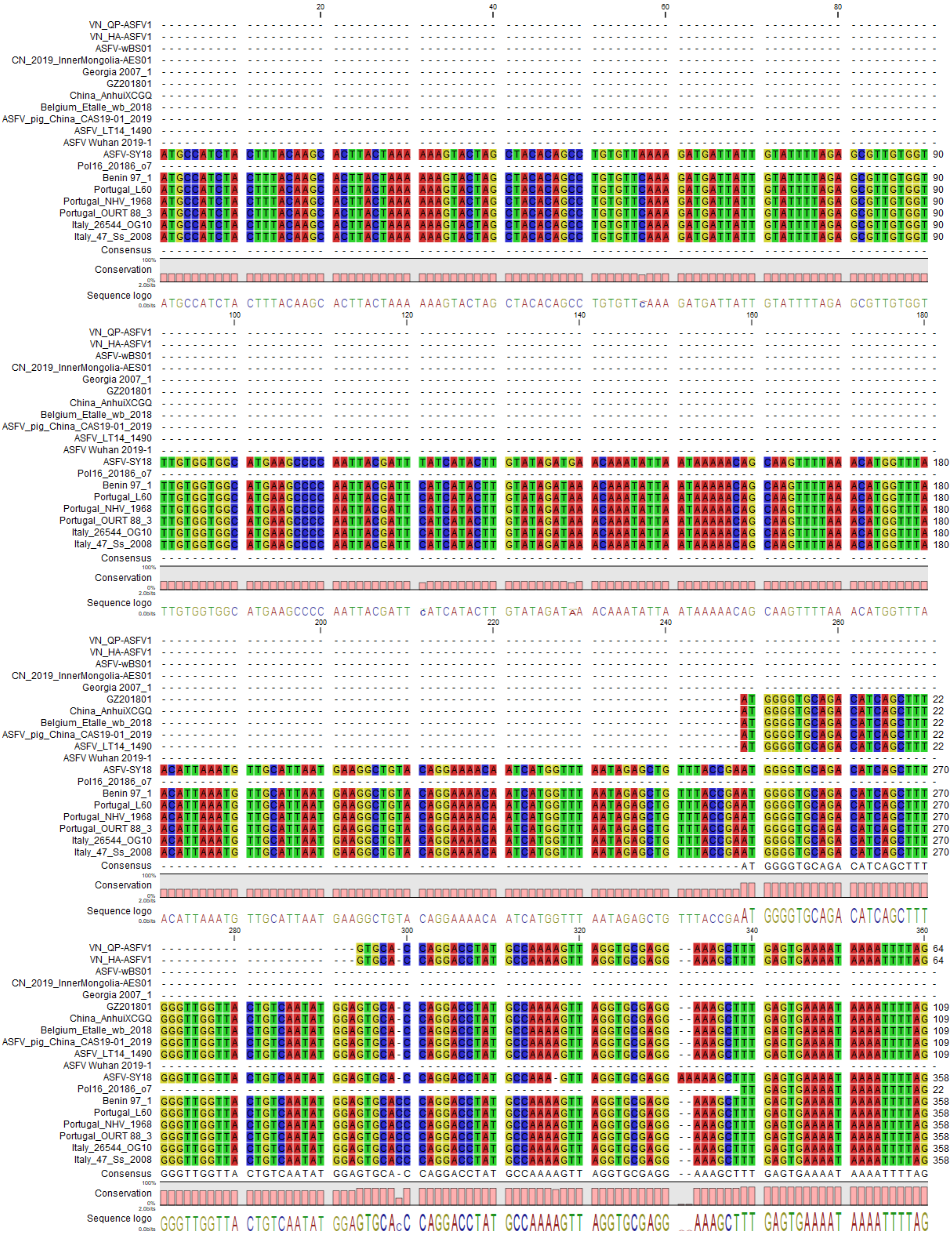

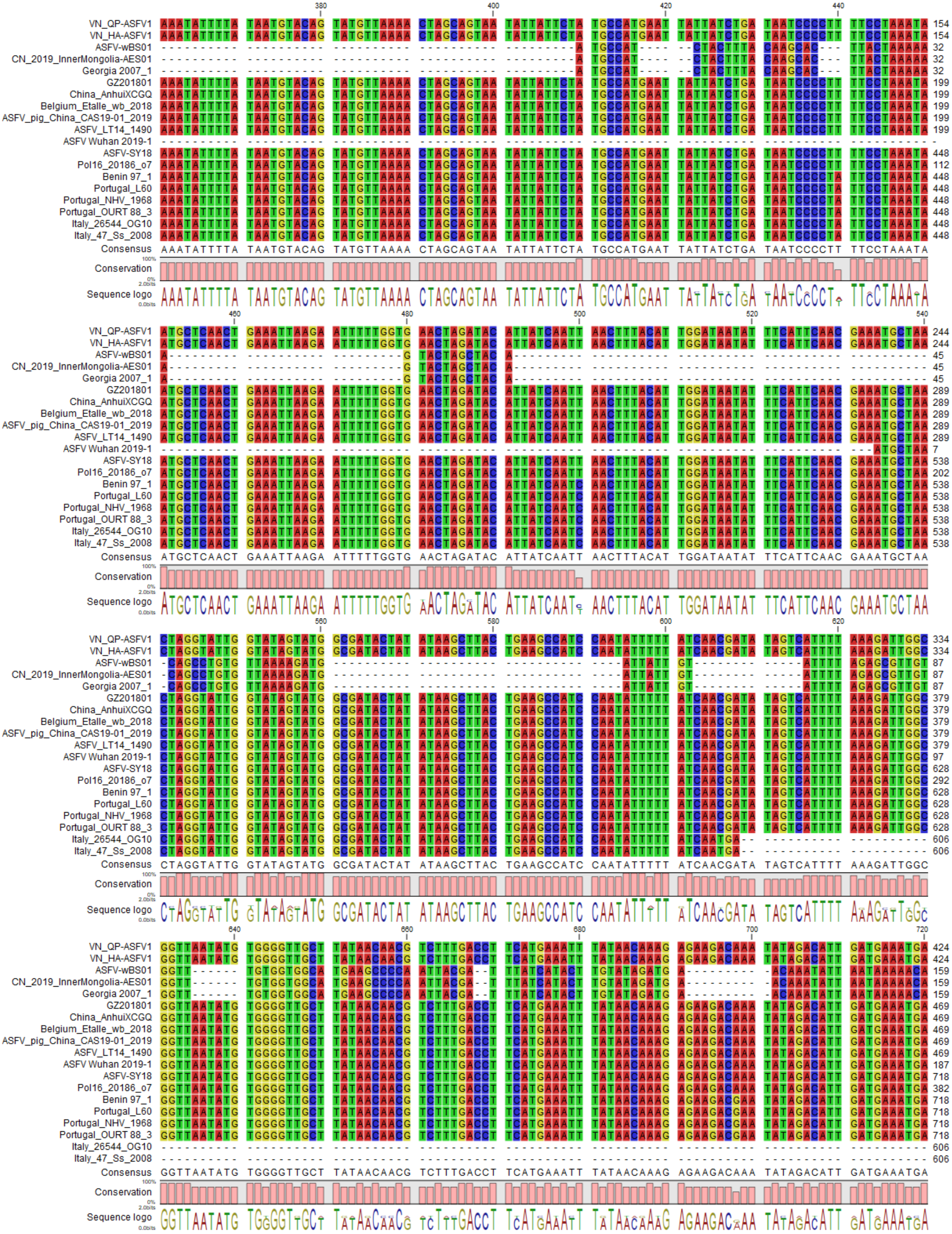
VN_QP-ASFV1
VN_HA-ASFV1
ASFV-wBS01
CN_2019_InnerMongolia-AES01
Georgia 2007_1
GZ201801
China_AnhuiXCGQ
Belgium_Etalle_wb_2018
ASFV_pig_China_CAS19-01_2019
ASFV_LT14_1490
ASFV Wuhan 2019-1
ASFV-SY18
Pol16_20186_o7
Benin 97_1
Portugal_L60
Portugal_NHV_1968
Portugal_OURT 88_3
Italy_26544_OG10
Italy_47_Ss_2008
Consensus
Conservation
Sequence logo
AAATATTTTA TAATGTACAG TATGTTAAAA CTAGCAGTAA TATTATTCTA TGCCATGAAT TATTATCTGA TAATCCCCTT TTCCTAAATA
ATGCTCAACT GAAATTAAGA ATTTTTGGTG AACTAGATAC ATTATCAATT AACTTTACAT TGGATAATAT TTCATTCAAC GAAATGCTAA
CTAGGTATTG GTATAGTATG GCGATACTAT ATAAGCTTAC TGAAGCCATC CAATATTTTT ATCAACGATA TAGTCATTTT AAAGATTGGC
GGTTAATATG TGGGGTTGCT TATAACAACG TCTTTGACCT TCATGAAATT TATAACAAAG AGAAGACAAA TATAGACATT GATGAAATGA

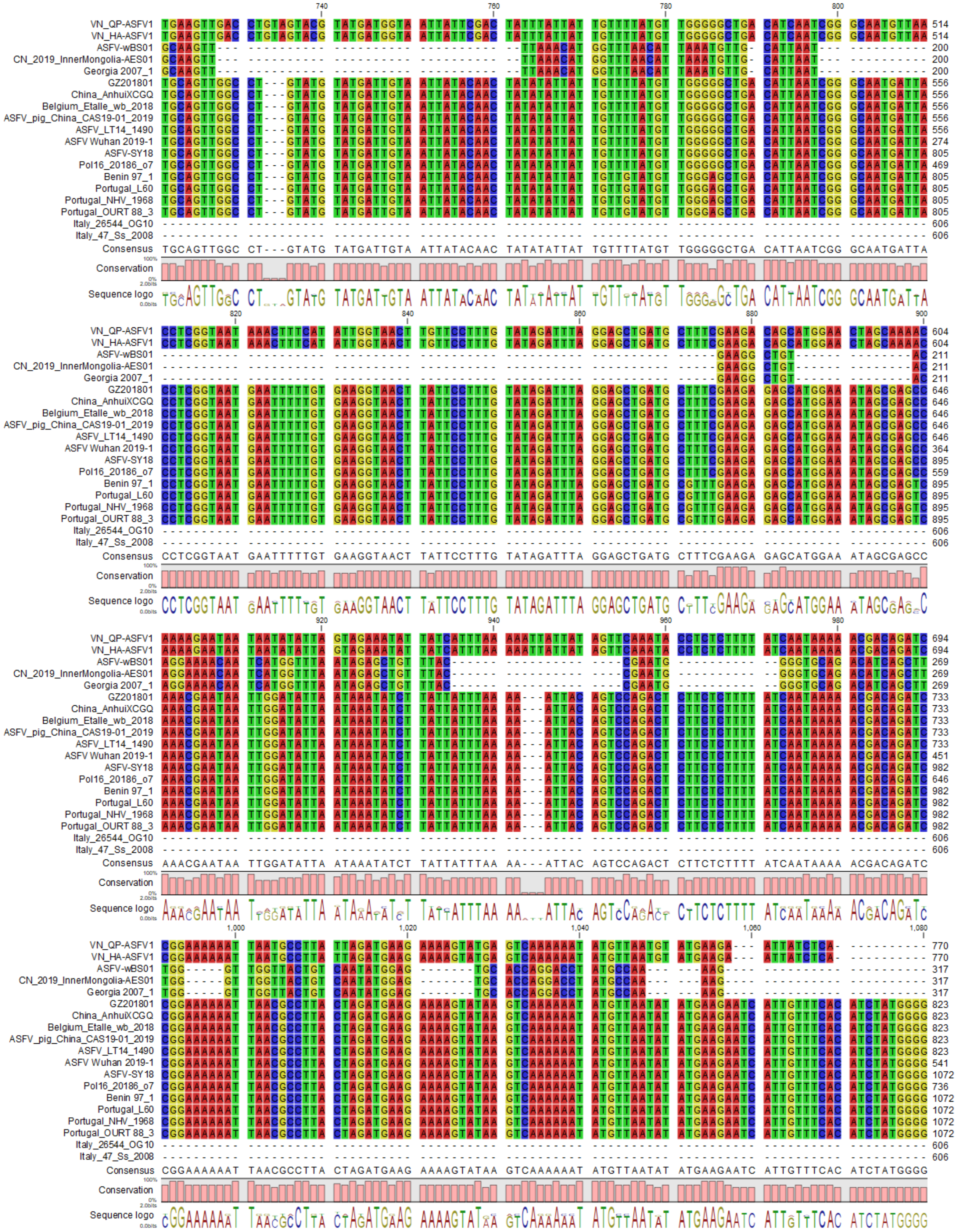

VN_QP-ASFV1
VN_HA-ASFV1
ASFV-wBS01
CN_2019_InnerMongolia-AES01
Georgia 2007_1
GZ201801
China_AnhuiXCGQ
Belgium_Etalle_wb_2018
ASFV_pig_China_CAS19-01_2019
ASFV_LT14_1490
ASFV Wuhan 2019-1
ASFV-SY18
Pol16_20186_o7
Benin 97_1
Portugal_L60
Portugal_NHV_1968
Portugal_OURT 88_3
Italy_26544_OG10
Italy_47_Ss_2008
Consensus
Conservation
Sequence logo
740
760
780
800
TGCAGTTGGC CT---GTATG TATGATTGTA ATTATACAAC TATATATTAT TGTTTTATGT TGGGGGCTGA CATTAATCGG GCAATGATTA
820
840
860
880
900
CCTCGGTAAT GAATTTTTGT GAAGGTAACT TATTCCTTTG TATAGATTTA GGAGCTGATG CTTTCGAAGA GAGCATGGAA ATAGCGAGCC
920
940
960
980
AAACGAATAA TTGGATATTA ATAAATATCT TATTATTTAA AA---ATTAC AGTCCAGACT CTTCTCTTTT ATCAATAAAA ACGACAGATC
1,000
1,020
1,040
1,060
1,080
CGGAAAAAAT TAACGCCTTA CTAGATGAAG AAAAGTATAA GTCAAAAAAT ATGTTAATAT ATGAAGAATC ATTGTTTCAC ATCTATGGGG

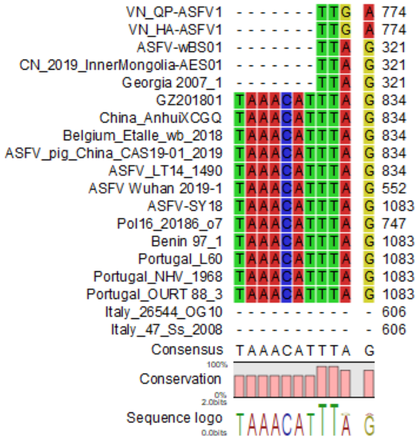
VN_QP-ASFV1 - - - - - - - T T G A 774
VN_HA-ASFV1 - - - - - - - T T G A 774
ASFV-wBS01 - - - - - - - T T A G 321
CN_2019_InnerMongolia-AES01 - - - - - - - T T A G 321
Georgia 2007_1 - - - - - - - T T A G 321
GZ201801 T A A A C A T T T A G 834
China_AnhuiXCGQ T A A A C A T T T A G 834
Belgium_Etalle_wb_2018 T A A A C A T T T A G 834
ASFV_pig_China_CAS19-01_2019 T A A A C A T T T A G 834
ASFV_LT14_1490 T A A A C A T T T A G 834
ASFV Wuhan 2019-1 T A A A C A T T T A G 552
ASFV-SY18 T A A A C A T T T A G 1083
Pol16_20186_o7 T A A A C A T T T A G 747
Benin 97_1 T A A A C A T T T A G 1083
Portugal_L60 T A A A C A T T T A G 1083
Portugal_NHV_1968 T A A A C A T T T A G 1083
Portugal_OURT 88_3 T A A A C A T T T A G 1083
Italy_26544_OG10 - - - - - - - - - - - 606
Italy_47_Ss_2008 - - - - - - - - - - - 606
Consensus T A A A C A T T T A G
Conservation
100%
0%
Sequence logo
2.0bits
0.0bits
T A A A C A T T T A G

(d) Sequence Alignment of MGF360-1L proteins

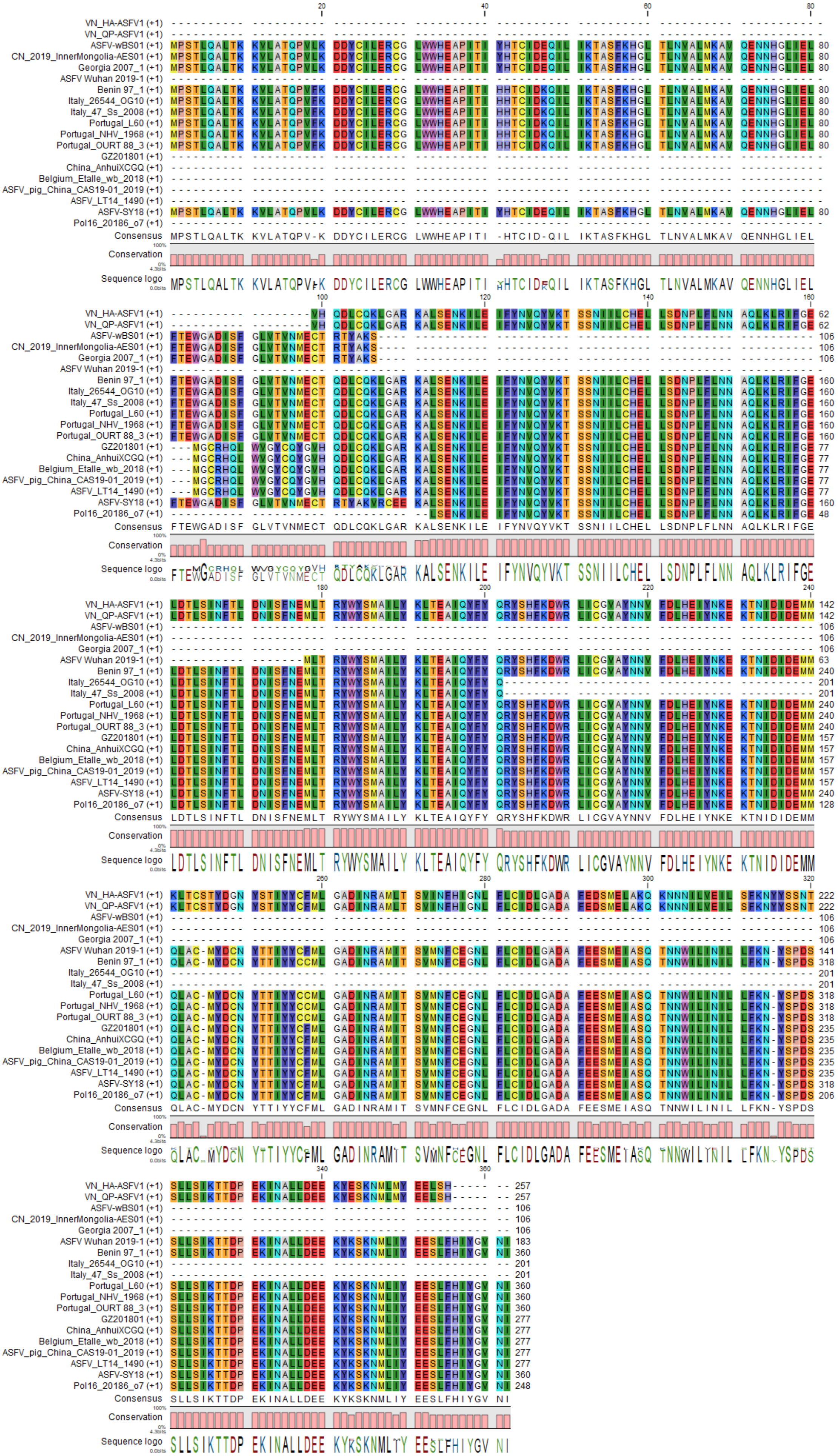

(e) Sequence Alignment of MGF110-1L nucleotides

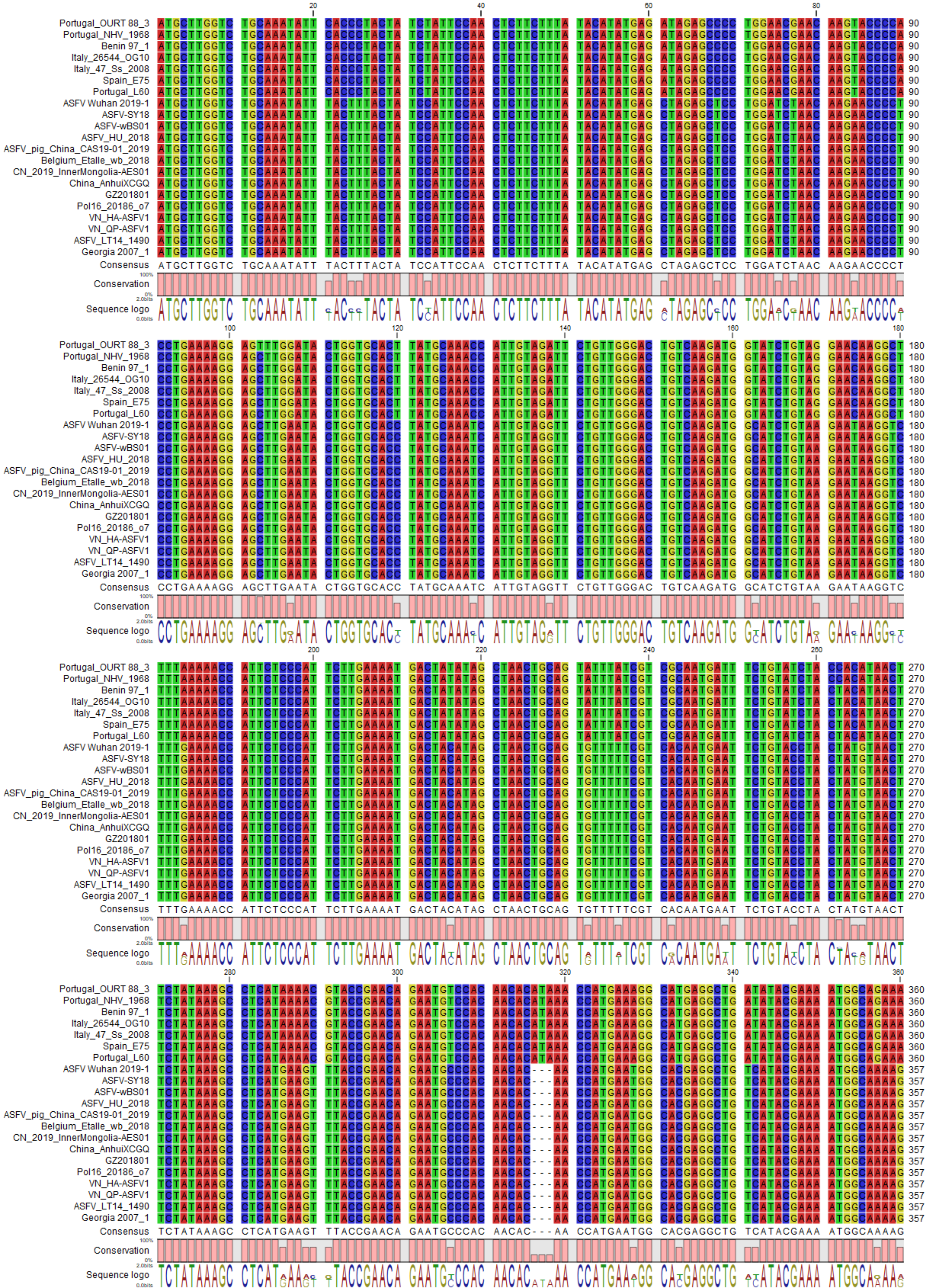

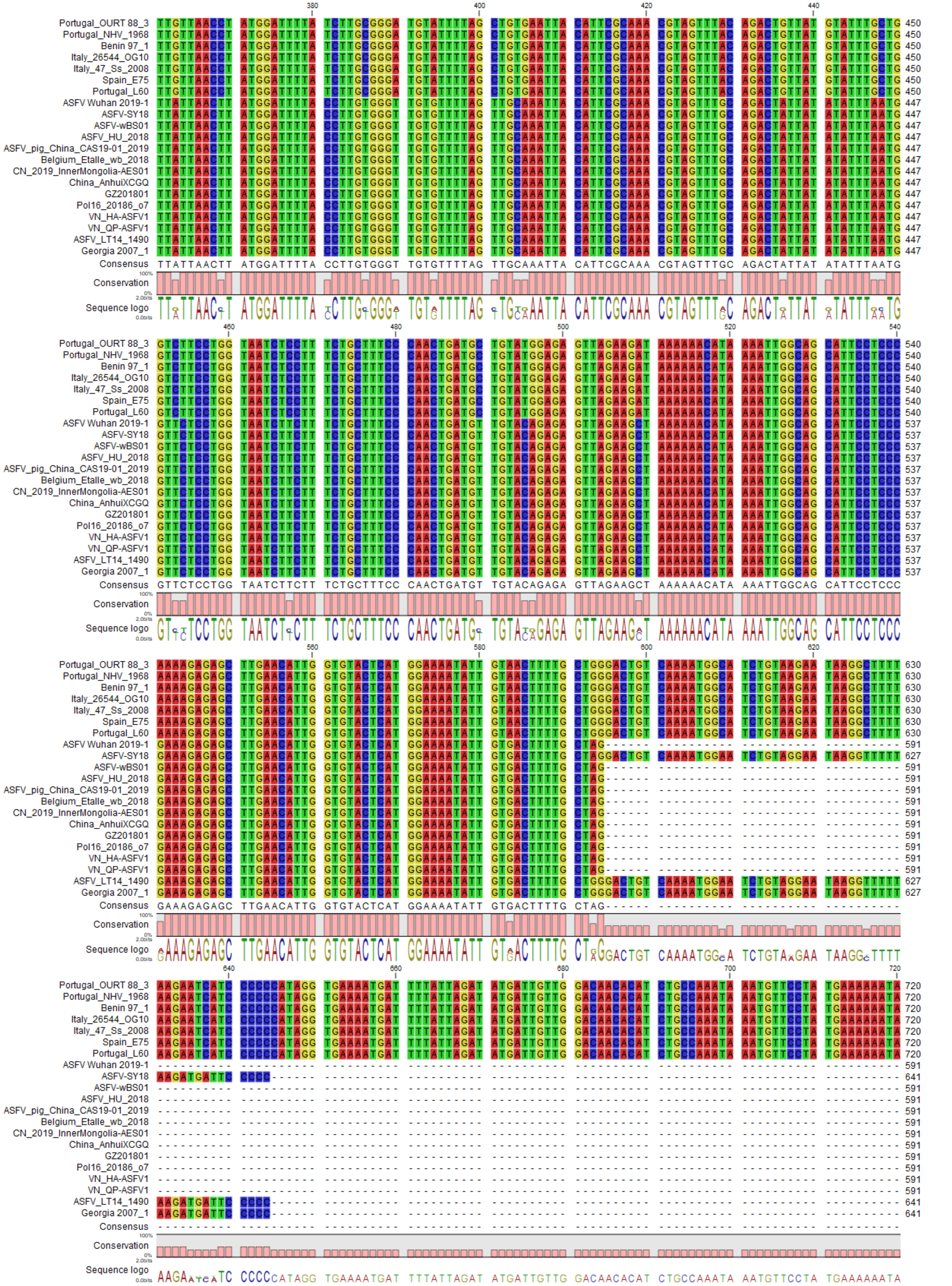

Portugal_OURT 88_3
Portugal_NHV_1968
Benin 97_1
Italy_26544_OG10
Italy_47_Ss_2008
Spain_E75
Portugal_L60
ASFV Wuhan 2019-1
ASFV-SY18
ASFV-wBS01
ASFV_HU_2018
ASFV_pig_China_CAS19-01_2019
Belgium_Etalle_wb_2018
CN_2019_InnerMongolia-AES01
China_AnhuiXCGQ
GZ201801
Pol16_20186_o7
VN_HA-ASFV1
VN_QP-ASFV1
ASFV_LT14_1490
Georgia 2007_1
Consensus
Conservation
Sequence logo
TTATTAACTT ATGGATTTTA CCTTGTGGGT TGTGTTTTAG TTGCAAATTA CATTCGCAAA CGTAGTTTGC AGACTATTAT ATATTTAATG
GTTCTCCTGG TAATCTTCTT TCTGCTTTCC CAACTGATGT TGTACAGAGA GTTAGAAGCT AAAAAACATA AAATTGGCAG CATTCCTCCC
GAAAGAGAGC TTGAACATTG GTGTACTCAT GGAAAATATT GTGACTTTTG CTAG

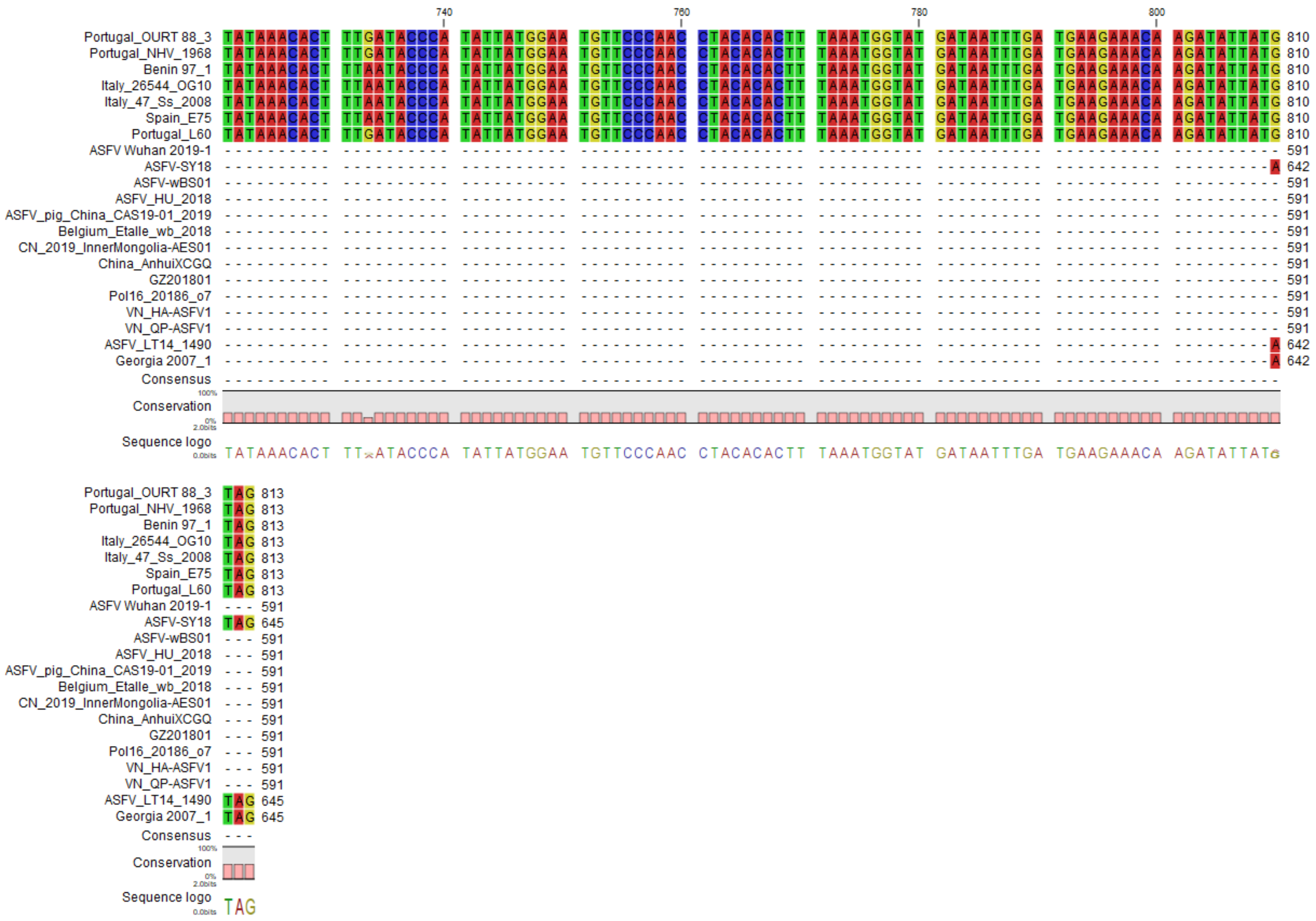
740
760
780
800
Portugal_OURT 88_3 TATAAACACT TTGATACCCA TATTATGGAA TGTTCCCAAC CTACACACTT TAAATGGTAT GATAATTTGA TGAAGAAACA AGATATTATG 810
Portugal_NHV_1968 TATAAACACT TTGATACCCA TATTATGGAA TGTTCCCAAC CTACACACTT TAAATGGTAT GATAATTTGA TGAAGAAACA AGATATTATG 810
Benin 97_1 TATAAACACT TTAATACCCA TATTATGGAA TGTTCCCAAC CTACACACTT TAAATGGTAT GATAATTTGA TGAAGAAACA AGATATTATG 810
Italy_26544_OG10 TATAAACACT TTAATACCCA TATTATGGAA TGTTCCCAAC CTACACACTT TAAATGGTAT GATAATTTGA TGAAGAAACA AGATATTATG 810
Italy_47_Ss_2008 TATAAACACT TTAATACCCA TATTATGGAA TGTTCCCAAC CTACACACTT TAAATGGTAT GATAATTTGA TGAAGAAACA AGATATTATG 810
Spain_E75 TATAAACACT TTAATACCCA TATTATGGAA TGTTCCCAAC CTACACACTT TAAATGGTAT GATAATTTGA TGAAGAAACA AGATATTATG 810
Portugal_L60 TATAAACACT TTGATACCCA TATTATGGAA TGTTCCCAAC CTACACACTT TAAATGGTAT GATAATTTGA TGAAGAAACA AGATATTATG 810
ASFV Wuhan 2019-1 591
ASFV-SY18 A 642
ASFV-wBS01 591
ASFV_HU_2018 591
ASFV_pig_China_CAS19-01_2019 591
Belgium_Etalle_wb_2018 591
CN_2019_InnerMongolia-AES01 591
China_AnhuiXCGQ 591
GZ201801 591
Pol16_20186_o7 591
VN_HA-ASFV1 591
VN_QP-ASFV1 591
ASFV_LT14_1490 A 642
Georgia 2007_1 A 642
Consensus
Conservation
Sequence logo
TATAAACACT TT ATACCCA TATTATGGAA TGTTCCCAAC CTACACACTT TAAATGGTAT GATAATTTGA TGAAGAAACA AGATATTATG
Portugal_OURT 88_3 TAG 813
Portugal_NHV_1968 TAG 813
Benin 97_1 TAG 813
Italy_26544_OG10 TAG 813
Italy_47_Ss_2008 TAG 813
Spain_E75 TAG 813
Portugal_L60 TAG 813
ASFV Wuhan 2019-1 591
ASFV-SY18 TAG 645
ASFV-wBS01 591
ASFV_HU_2018 591
ASFV_pig_China_CAS19-01_2019 591
Belgium_Etalle_wb_2018 591
CN_2019_InnerMongolia-AES01 591
China_AnhuiXCGQ 591
GZ201801 591
Pol16_20186_o7 591
VN_HA-ASFV1 591
VN_QP-ASFV1 591
ASFV_LT14_1490 TAG 645
Georgia 2007_1 TAG 645
Consensus
Conservation
Sequence logo
TAG

(f) Sequence Alignment of MGF110-1L proteins

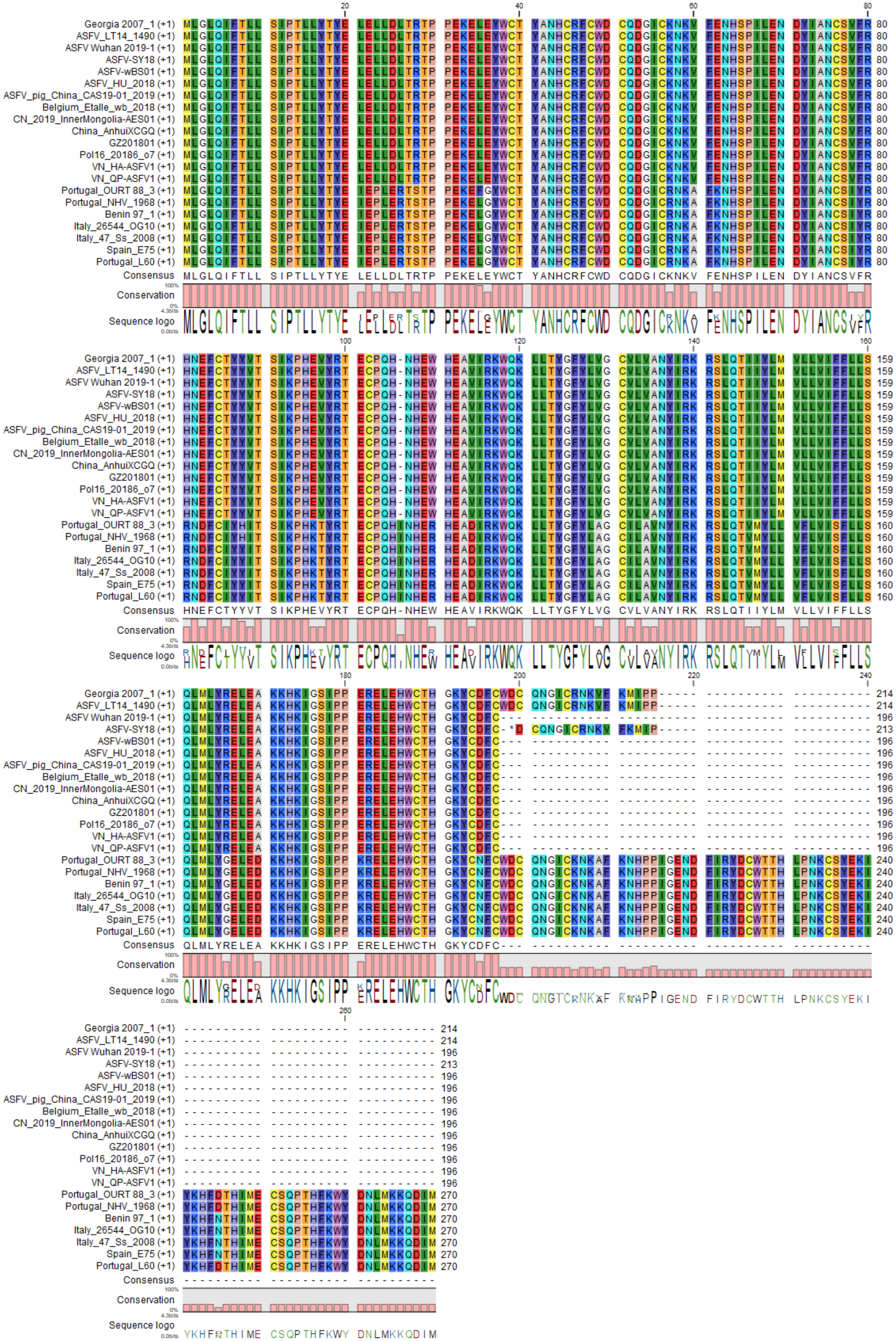

Supplement: Supplementary file 1 [file viruses-15-01945-s001.zip › Figure S1.pdf]
