# Supplementary material for: A Whole-Genome Analysis of the African Swine Fever Virus That Circulated during the First Outbreak in Vietnam in 2019 and Subsequently in 2022
Source: Viruses. 2023 Sep 18;15(9):1945. doi: 10.3390/v15091945 (PMC10537361; doi:10.3390/v15091945)
Supplement: Supplementary file 1 [file viruses-15-01945-s001.zip › Table S1.pdf]

Table S1. List of genetic information for representative 28 ASFV strains used in this study

	Name	GeneBank No	Length(bp)	p72Genotype	Host	origin(country)	year
1	VN/HY/2019-ASFV1	MT872723	189,113	II	pig	Vietnam	2019
2	VN/QP/2019-ASFV1	MT882025	189,081	II	pig	Vietnam	2019
3	VN/HY/2022-ASFV2	OR227304	189,607	II	pig	Vietnam	2022
4	China/AnhuiXCGQ	MK128995	189,354	II	pig	China	2018
5	POL/2015/Podlaskie	MH681419	189,394	II	pig	Poland	2015
6	Georgia 2007/1	FR682468	189,344	II	pig	Georgia	2007
7	Estonia 2014	LS478113	182,446	II	Wild boar	Estonia	2014
8	Russia/Odintsovo_02/14	KP843857	189,333	II	Wild boar	Russia	2014
9	Spain/E75	FN557520	181,187	I	pig	Spain	2010
10	SA/Mkuzi1979	AY261362	192,714	I/VII	Tick	South Africa	1979
11	Portugal/OURT 88/3	AM712240	171,719	I	Tick	Portugal	1988
12	Benin 97/1	AM712239	182,284	I	pig	Benin	1997
13	Portugal/L60	KM262844	182,362	I	pig	Portugal	1960
14	Italy/26544/OG10	KM102979	182,906	I	pig	Italy	2010
15	Portugal/NHV/1968	KM262845	172,051	I	pig	Portugal	1968
16	Italy/47/Ss/2008	KX354450	184,638	I	pig	Italy	2008
17	Spain/BA71	KP055815	180,365	I	pig	Spain	1971
18	ASFV-SY18	MH766894	188,643	II	pig	China	2018
19	Kashino 04/13	KJ747406	189,387	II	Wild boar	Russia	2013
20	ASFV-wBS01	MK645909	189,394	II	Wild boar	China	2018
21	Belgium/Etalle/wb/2018	MK543947	190,202	II	Wild boar	Belgium	2018
22	ASFV/LT14/1490	MK628478	189,399	II	pig	Lithuania	2014
23	ASFV/pig/China/CAS19-01/2019	MN172368	189,405	II	pig	China	2019
24	ASFV_HU_2018	MN715134	190,601	II	Wild boar	Hungary	2018
25	ASFV Wuhan 2019-1	MN393476	190,576	II	pig	China	2019
26	CN/2019/InnerMongolia-AES01	MK940252	189,403	II	Wild boar	China	2019
27	Pol16_20186_o7	MG939583	189,401	II	pig	Poland	2018
28	GZ201801	MT496893	189,393	II	pig	China	2018
