# Supplementary material for: A Whole-Genome Analysis of the African Swine Fever Virus That Circulated during the First Outbreak in Vietnam in 2019 and Subsequently in 2022
Source: Viruses. 2023 Sep 18;15(9):1945. doi: 10.3390/v15091945 (PMC10537361; doi:10.3390/v15091945)
Supplement: Supplementary file 1 [file viruses-15-01945-s001.zip › Table S2.pdf]

Table S2. Whole-genome average nucleotide identity (ANI) and alignment percentage (AP) of 27 ASFV Strains.

		SA/Mkuzi1979	ASFV_HU_2018	ASFV Wuhan 2019-1	ASFV-SY18	ASFV-wBS01	ASFV/LT14/1490	ASFV/pig/China/CAS19-01/2019	Belgium/Etalle/wb/2018	Benin 97/1	CN/2019/InnerMongolia-AES01	China/AnhuiXCGQ	Estonia 2014	GZ201801	Georgia 2007/1	Italy/26544/OG10	Italy/47/Ss/2008	Kashino 04/13	POL/2015/Podlaskie	Pol16_20186_o7	Portugal/L60	Portugal/NHV/1968	Portugal/OURT 88/3	Russia/Odintsovo_02/14	Spain/BA71	Spain/E75	VN/HY/2019-ASFV1	VN/QP/2019-ASFV1	VN/HY/2022-ASFV2
		1	2	3	4	5	6	7	8	9	10	11	12	13	14	15	16	17	18	19	20	21	22	23	24	25	26	27	28
SA/Mkuzi1979	1		96.89	96.89	96.86	96.88	96.89	96.89	96.89	98.00	96.88	96.89	96.95	96.88	96.89	98.03	98.03	96.89	96.89	96.89	98.03	97.80	97.83	96.86	98.09	97.91	96.83	96.83	96.87
ASFV_HU_2018	2	95.11		100.00	99.98	99.99	99.99	100.00	100.00	97.18	99.99	100.00	99.92	99.99	99.99	97.19	97.19	99.98	99.99	99.99	97.27	97.14	97.22	99.98	97.10	97.07	99.93	99.93	99.92
ASFV Wuhan 2019-1	3	95.11	99.66		99.98	99.99	99.99	100.00	100.00	97.18	99.99	100.00	99.93	99.99	99.99	97.19	97.19	99.98	99.99	99.99	97.27	97.14	97.22	99.98	97.10	97.08	99.93	99.93	99.92
ASFV-SY18	4	95.42	99.46	99.46		99.98	99.97	99.98	99.98	97.15	99.97	99.98	99.91	99.97	99.98	97.16	97.16	99.97	99.97	99.98	97.24	97.13	97.21	99.97	97.08	97.05	99.92	99.92	99.90
ASFV-wBS01	5	95.41	99.47	99.47	99.81		99.99	99.99	99.99	97.17	99.99	99.99	99.92	99.99	99.99	97.19	97.19	99.97	99.98	99.99	97.27	97.13	97.22	99.97	97.09	97.07	99.93	99.93	99.91
ASFV/LT14/1490	6	95.41	99.47	99.47	99.87	99.82		99.99	99.99	97.17	99.98	99.99	99.86	99.98	99.99	97.19	97.19	99.97	100.00	99.99	97.27	97.14	97.22	99.97	97.10	97.07	99.93	99.93	99.91
ASFV/pig/China/CAS19-01/2019	7	95.41	99.47	99.48	99.87	99.82	99.99		100.00	97.18	99.99	99.99	99.87	99.99	99.99	97.19	97.19	99.97	99.99	99.99	97.27	97.14	97.22	99.98	97.10	97.07	99.93	99.93	99.92
Belgium/Etalle/wb/2018	8	95.21	99.28	99.29	99.66	99.61	99.78	99.79		97.18	99.99	99.99	99.92	99.99	100.00	97.19	97.19	99.98	99.99	99.99	97.27	97.14	97.22	99.98	97.10	97.07	99.93	99.93	99.92
Benin 97/1	9	95.10	94.51	94.52	94.82	94.81	94.81	94.81	94.61		97.17	97.17	97.13	97.17	97.18	99.94	99.94	97.16	97.18	97.17	99.92	99.83	99.83	97.06	99.86	99.93	97.02	97.02	97.04
CN/2019/InnerMongolia-AES01	10	95.41	99.47	99.48	99.77	99.78	99.78	99.78	99.58	94.81		99.99	99.91	99.98	99.99	97.18	97.18	99.97	99.98	99.99	97.26	97.13	97.21	99.97	97.09	97.07	99.93	99.93	99.91
China/AnhuiXCGQ	11	95.41	99.47	99.48	99.87	99.82	99.99	100.00	99.78	94.81	99.79		99.87	99.99	99.99	97.19	97.19	99.97	99.99	99.99	97.27	97.14	97.22	99.98	97.10	97.07	99.93	99.93	99.91
Estonia 2014	12	91.78	94.53	94.65	94.39	94.34	94.47	94.47	94.42	93.77	94.31	94.47		99.86	99.81	97.13	97.13	99.90	99.86	99.87	97.16	97.29	97.35	99.90	97.13	97.23	99.99	99.99	99.97
GZ201801	13	95.41	99.47	99.48	99.87	99.82	99.99	100.00	99.78	94.81	99.79	100.00	94.47		99.99	97.18	97.18	99.97	99.98	99.99	97.26	97.13	97.21	99.97	97.10	97.06	99.92	99.93	99.91
Georgia 2007/1	14	95.11	99.41	99.42	99.50	99.51	99.51	99.51	99.32	94.52	99.47	99.51	94.87	99.51		97.19	97.19	99.98	99.99	99.99	97.27	97.14	97.23	99.98	97.10	97.08	99.93	99.93	99.91
Italy/26544/OG10	15	94.94	94.35	94.36	94.65	94.64	94.64	94.64	94.45	99.61	94.64	94.65	93.61	94.65	94.35		99.98	97.17	97.19	97.19	99.94	99.86	99.87	97.16	99.87	99.96	97.01	97.01	97.04
Italy/47/Ss/2008	16	94.48	93.90	93.90	94.20	94.19	94.19	94.19	94.00	99.14	94.19	94.19	93.17	94.19	93.90	99.37		97.17	97.19	97.19	99.94	99.86	99.87	97.16	99.87	99.95	97.01	97.01	97.04
Kashino 04/13	17	95.31	98.09	98.10	98.40	98.40	98.40	98.40	98.20	94.72	98.40	98.40	92.90	98.40	98.10	94.59	94.14		99.97	99.97	97.25	97.14	97.22	99.97	97.12	97.06	99.91	99.91	99.90
POL/2015/Podlaskie	18	95.41	99.47	99.47	99.86	99.82	99.99	99.99	99.78	94.81	99.78	99.99	94.47	99.99	99.51	94.64	94.19	98.40		99.99	97.27	97.14	97.22	99.97	97.10	97.07	99.92	99.92	99.91
Pol16/20186/o7	19	95.41	99.47	99.48	99.87	99.82	99.99	100.00	99.79	94.81	99.78	100.00	94.48	100.00	99.51	94.64	94.19	98.40	99.99		97.27	97.14	97.22	99.98	97.10	97.07	99.93	99.93	99.91
Portugal/L60	20	95.08	93.94	93.95	94.25	94.24	94.24	94.23	94.04	99.80	94.24	94.24	93.75	94.24	93.95	99.60	99.13	94.21	94.24	94.24		99.95	99.90	97.25	99.91	99.96	96.99	96.99	97.02
Portugal/NHV/1968	21	93.30	92.37	92.38	92.64	92.65	92.65	92.65	92.46	94.22	92.65	92.65	89.05	92.65	92.37	93.95	93.52	92.61	92.65	92.65	93.69		99.99	97.13	99.89	99.85	97.16	97.16	97.19
Portugal/OURT 88/3	22	93.29	92.34	92.35	92.62	92.62	92.62	92.62	92.44	94.31	92.62	92.62	89.12	92.62	92.34	93.99	93.56	92.58	92.62	92.62	94.16	99.89		97.21	99.83	99.89	97.22	97.22	97.26
Russia/Odintsovo_02/14	23	95.43	99.45	99.45	99.87	99.80	99.86	99.86	99.65	95.38	99.76	99.86	94.38	99.86	99.49	94.66	94.21	98.39	99.86	99.86	94.25	92.64	92.61		97.09	97.04	99.91	99.91	99.89
Spain/BA71	24	92.87	93.37	93.38	93.67	93.66	93.66	93.66	93.47	96.73	93.66	93.66	92.01	93.66	93.38	96.55	96.10	93.23	93.66	93.66	96.87	93.49	93.39	93.68		99.76	97.39	97.39	97.32
Spain/E75	25	94.85	94.26	94.26	94.56	94.55	94.55	94.55	94.35	99.41	94.55	94.55	93.49	94.55	94.26	99.20	98.73	94.48	94.55	94.55	99.53	93.94	93.84	94.56	97.01		97.09	97.09	97.12
VN/HY/2019-ASFV1	26	95.48	99.39	99.40	99.69	99.70	99.70	99.70	99.49	95.43	99.70	99.71	94.38	99.71	99.39	95.26	94.81	98.32	99.70	99.70	95.41	92.58	92.06	99.68	92.06	94.62		100.00	99.98
VN/QP/2019-ASFV1	27	95.49	99.40	99.40	99.70	99.71	99.71	99.72	99.50	95.44	99.71	99.72	94.39	99.72	99.40	95.27	94.82	98.33	99.71	99.71	95.42	92.58	92.07	99.69	92.07	94.63	99.80		99.98
VN/HY/2022-ASFV2	28	95.36	99.24	99.25	99.54	99.55	99.55	99.55	99.34	95.31	99.55	99.56	94.24	99.56	99.24	95.14	94.69	98.17	99.55	99.55	95.29	92.49	91.97	99.53	92.50	94.50	99.64	99.64	

AP ANI

* The area with yellow ANI on the table indicates average nucleotide identity(ANI), and the area with green AP indicates alignment percentage(AP).
