# Supplementary material for: A Whole-Genome Analysis of the African Swine Fever Virus That Circulated during the First Outbreak in Vietnam in 2019 and Subsequently in 2022
Source: Viruses. 2023 Sep 18;15(9):1945. doi: 10.3390/v15091945 (PMC10537361; doi:10.3390/v15091945)
Supplement: Supplementary file 1 [file viruses-15-01945-s001.zip › Table S3.pdf]

Table S3. Sequence identities of 28 ORFs compared VN/HY/2019-ASFV1 and VN/QP/2019-ASFV1 with 22 particular ASFV strains

(a) Sequence identities of nucelotides

ASFV strain name	O174L	NP419L	MGF_505-9	MGF_505-5	GF_360-2	MGF_360-1	GF_360-1	GF_360-1	MGF_110-1	GF_110-1	GF_110-13	GF_110-1	M1249L
**VN/QP/2019-ASFV1**	100.00	99.92	100.00	100.00	100.00	100.00	100.00	100.00	100.00	99.13	100.00	100.00	100.00
**VN/HY/2019-ASFV1**	100.00	100.00	100.00	100.00	100.00	100.00	100.00	100.00	100.00	100.00	100.00	100.00	100.00
**Spain_E75**	97.33	98.65	97.50	98.73	82.44	82.41	98.11	98.49	89.02	95.68	99.95	98.95	98.82
**Spain_BA71**	97.52	98.65	97.50	98.86	77.96	92.18	98.20	98.49	100.00	22.41	12.36	12.36	98.85
**Portugal_OURT_88_3**	97.33	98.65	97.50	98.73	77.96	92.18	98.11	2.43	88.68	83.79	69.18	69.18	98.88
**Portugal_NHV_1968**	97.33	98.65	97.50	98.73	77.96	92.18	98.11	2.43	88.85	83.84	69.18	69.18	98.88
**Portugal_L60**	97.33	98.65	97.50	98.73	77.96	92.18	98.20	98.49	89.02	95.68	97.27	97.27	98.85
**Pol16_20186_o7**	100.00	100.00	100.00	99.93	100.00	93.04	99.91	99.88	100.00	99.15	99.58	99.58	100.00
**Italy_47_Ss_2008**	97.33	98.65	97.43	98.73	77.96	92.06	97.92	98.37	89.02	94.82	97.27	97.27	98.82
**Italy_26544_OG10**	97.33	98.65	97.43	98.73	77.96	92.06	98.01	98.49	89.02	95.11	97.48	97.48	98.80
**GZ201801**	99.81	100.00	100.00	100.00	100.00	93.04	100.00	99.88	100.00	100.00	99.58	99.58	100.00
**Georgia_2007_1**	100.00	99.92	99.93	100.00	100.00	93.04	100.00	99.88	99.66	99.13	99.58	99.58	100.00
**Estonia_2014**	100.00	100.00	100.00	100.00	100.00	89.26	100.00	99.88	10.32	81.38	32.70	32.70	100.00
**CN_2019_InnerMongolia-AES01**	100.00	100.00	100.00	100.00	100.00	93.04	100.00	99.88	100.00	100.00	99.58	99.58	99.95
**China_AnhuiXCGQ**	99.43	100.00	100.00	100.00	100.00	92.06	100.00	99.88	100.00	100.00	99.58	99.58	100.00
**Benin_97_1**	97.33	98.65	97.50	98.73	77.96	92.18	97.92	98.49	89.02	94.82	97.27	97.27	98.85
**Belgium_Etalle_wb_2018**	100.00	100.00	100.00	100.00	100.00	93.04	100.00	99.88	100.00	99.13	99.58	99.58	100.00
**ASFV-wBS01**	100.00	100.00	100.00	100.00	100.00	93.04	100.00	99.88	100.00	99.15	99.58	99.58	99.97
**ASFV_Wuhan_2019-1**	100.00	100.00	100.00	100.00	99.91	93.04	100.00	100.00	100.00	98.85	99.16	99.16	100.00
**ASFV_pig_China_CAS19-01_2019**	100.00	100.00	100.00	100.00	100.00	93.04	100.00	99.88	100.00	99.15	99.58	99.58	100.00
**ASFV_LT14_1490**	100.00	99.92	99.93	100.00	100.00	93.04	100.00	99.88	99.66	99.13	98.32	98.32	100.00
**ASFV_HU_2018**	100.00	100.00	100.00	100.00	100.00	93.04	100.00	99.88	100.00	99.13	99.58	99.58	100.00

ASFV strain name	L11L	KP177R	K145R	I73R	I267L	I196L	EP153R	E199L	DP238L	D1133L	CP204L	CP123L	C84L	C717R	C315R
**VN/QP/2019-ASFV1**	100.00	100.00	100.00	100.00	100.00	100.00	100.00	99.83	100.00	100.00	100.00	100.00	100.00	100.00	100.00
**VN/HY/2019-ASFV1**	100.00	100.00	100.00	100.00	100.00	100.00	100.00	100.00	100.00	100.00	100.00	100.00	100.00	100.00	100.00
**Spain_E75**	98.22	97.56	98.85	96.80	97.63	93.26	56.81	99.33	98.88	99.14	97.77	95.69	99.22	99.07	99.05
**Spain_BA71**	98.23	97.56	98.85	96.34	97.63	93.26	56.81	99.83	98.88	99.17	97.77	95.69	99.21	100.00	99.15
**Portugal_OURT_88_3**	97.87	97.56	98.85	96.80	97.63	93.26	56.81	99.33	98.88	99.18	97.60	95.69	99.22	99.12	99.16
**Portugal_NHV_1968**	97.87	97.56	98.85	96.80	97.63	93.26	56.81	99.33	98.88	99.18	96.92	95.69	99.22	99.12	99.16
**Portugal_L60**	97.87	97.56	98.85	96.80	97.63	93.26	56.81	99.50	98.88	99.18	97.77	95.69	99.22	99.12	99.16
**Pol16_20186_o7**	100.00	100.00	99.77	100.00	100.00	100.00	100.00	99.67	100.00	100.00	100.00	100.00	100.00	100.00	100.00
**Italy_47_Ss_2008**	98.22	97.56	98.63	96.80	97.63	93.26	56.81	99.50	98.88	99.18	97.77	95.69	99.22	99.03	99.05
**Italy_26544_OG10**	98.22	97.56	98.63	96.80	97.63	93.26	56.81	99.50	98.88	99.18	97.77	95.69	99.22	99.03	99.16
**GZ201801**	100.00	100.00	100.00	100.00	100.00	100.00	100.00	99.83	100.00	100.00	100.00	100.00	100.00	100.00	100.00
**Georgia_2007_1**	100.00	100.00	100.00	100.00	99.87	100.00	100.00	99.83	100.00	100.00	100.00	100.00	100.00	100.00	100.00
**Estonia_2014**	100.00	33.52	100.00	100.00	100.00	100.00	100.00	99.83	100.00	100.00	100.00	100.00	100.00	100.00	100.00
**CN_2019_InnerMongolia-AES01**	100.00	100.00	100.00	98.63	100.00	100.00	100.00	99.83	100.00	100.00	99.83	99.73	100.00	100.00	100.00
**China_AnhuiXCGQ**	100.00	100.00	100.00	100.00	100.00	100.00	100.00	99.83	100.00	100.00	100.00	100.00	100.00	100.00	100.00
**Benin_97_1**	97.87	97.56	98.85	96.80	97.51	93.26	56.81	99.50	98.74	99.18	97.77	95.69	99.22	99.07	99.16
**Belgium_Etalle_wb_2018**	100.00	100.00	100.00	100.00	100.00	100.00	100.00	99.83	100.00	100.00	100.00	100.00	100.00	100.00	100.00
**ASFV-wBS01**	99.65	100.00	100.00	100.00	100.00	99.67	99.79	99.83	99.58	99.97	100.00	100.00	100.00	100.00	100.00
**ASFV_Wuhan_2019-1**	100.00	100.00	100.00	100.00	100.00	100.00	98.75	99.83	100.00	100.00	100.00	100.00	100.00	99.95	100.00
**ASFV_pig_China_CAS19-01_2019**	100.00	100.00	100.00	100.00	100.00	100.00	100.00	99.83	100.00	100.00	100.00	100.00	100.00	100.00	100.00
**ASFV_LT14_1490**	100.00	100.00	100.00	100.00	99.88	100.00	100.00	99.67	100.00	100.00	100.00	100.00	100.00	100.00	100.00
**ASFV_HU_2018**	100.00	100.00	100.00	100.00	100.00	100.00	100.00	99.83	100.00	100.00	100.00	100.00	100.00	100.00	99.89

(b) Sequence identities of proteins

ASFV strain name	O174L	NP419L	MGF_505-9	MGF_505-5	GF_360-2	MGF_360-1	GF_360-1	GF_360-1	MGF_110-1	GF_110-1	GF_110-13	GF_110-1	M1249L
**VN/QP/2019-ASFV1**	100	99.76	100	100	100	100	100	100	100	100	100	100	100
**VN/HY/2019-ASFV1**	100	100	100	100	100	100	100	100	100	100	100	100	100
**Spain_E75**	96.57	99.05	97.04	98.6	81.48	73.71	96.76	97.91	83.25	92	98.52	98.1	98.71
**Spain_BA71**	96.57	99.05	97.04	98.6	81.48	90.08	98.02	97.91	83.25	51.79	45.45	46.73	99.28
**Portugal_OURT_88_3**	96.57	99.05	97.04	98.4	71.95	90.08	96.76	36.59	82.23	76.7	93.6	69.03	99.36
**Portugal_NHV_1968**	96.57	99.05	97.04	98.4	71.95	90.08	96.76	36.59	82.74	76.92	93.65	69.03	99.36
**Portugal_L60**	96.57	99.05	97.04	98.6	71.95	90.08	94.92	97.91	83.25	92	97.04	98.06	98.8
**Pol16_20186_o7**	100	100	100	99.8	100	91.08	100	98.95	100	100	100	100	100
**Italy_47_Ss_2008**	96.57	99.05	97.04	98.6	81.48	89.68	98.94	97.56	83.25	91.92	97.62	95.51	99.28
**Italy_26544_OG10**	96.57	99.05	97.04	98.6	81.48	89.68	98.02	97.91	83.25	91.92	97.62	96.15	99.28
**GZ201801**	99.43	100	100	100	100	91.08	100	98.95	100	100	100	100	100
**Georgia_2007_1**	100	99.76	99.8	100	100	91.08	100	98.95	100	100	100	100	100
**Estonia_2014**	100	100	100	100	100	83.2	100	98.95	50.67	73.08	100	100	100
**CN_2019_InnerMongolia-AES01**	100	100	100	100	100	91.08	100	98.95	100	100	99.19	100	99.92
**China_AnhuiXCGQ**	98.29	100	100	100	100	91.08	100	98.95	100	100	100	100	100
**Benin_97_1**	96.57	99.05	97.04	98.6	81.48	90.08	94.55	97.91	83.25	91.92	97.04	98.06	99.28
**Belgium_Etalle_wb_2018**	100	100	100	100	100	91.08	100	98.95	100	100	100	100	100
**ASFV-wBS01**	100	100	100	100	100	91.08	100	98.95	100	100	100	100	99.92
**ASFV_Wuhan_2019-1**	100	100	100	100	99.72	91.08	100	100	100	100	100	100	100
**ASFV_pig_China_CAS19-01_2019**	100	100	100	100	100	91.08	100	98.95	100	100	100	100	100
**ASFV_LT14_1490**	100	99.76	99.8	100	100	91.08	100	98.95	100	100	98.52	100	100
**ASFV_HU_2018**	100	100	100	100	100	91.08	100	98.95	100	100	100	100	100

ASFV strain name	L11L	KP177R	K145R	I73R	I267L	I196L	EP153R	E199L	DP238L	D1133L	CP204L	CP123L	C84L	C717R	C315R
**VN/QP/2019-ASFV1**	100	100	100	100	100	100	100	99.5	100	100	100	100	100	100	100
**VN/HY/2019-ASFV1**	100	100	100	100	100	100	100	100	100	100	100	100	100	100	100
**Spain_E75**	95.74	98.31	100	98.59	97.39	90.16	51.38	98.5	98.57	99.82	97.95	95.97	98.82	100	100
**Spain_BA71**	95.74	98.31	100	98.59	97.39	90.16	51.38	99.5	98.57	99.91	97.95	95.97	98.82	100	100
**Portugal_OURT_88_3**	91.4	98.31	100	98.59	97.39	90.16	51.38	98	98.57	99.91	97.44	95.97	98.82	100	100
**Portugal_NHV_1968**	91.4	98.31	100	98.59	97.39	90.16	51.38	98	98.57	99.91	97.34	95.97	98.82	100	100
**Portugal_L60**	94.62	98.31	100	98.59	97.39	90.16	51.38	98.5	98.57	99.91	97.95	95.97	98.82	100	100
**Pol16_20186_o7**	100	100	100	100	100	100	100	99	100	100	100	100	100	100	100
**Italy_47_Ss_2008**	95.74	98.31	99.32	98.59	97.39	90.16	51.38	98.5	98.57	99.91	97.95	95.97	98.82	99.86	100
**Italy_26544_OG10**	95.74	98.31	99.32	98.59	97.39	90.16	51.38	98.5	98.57	99.91	97.95	95.97	98.82	99.86	100
**GZ201801**	100	100	100	100	100	100	100	99.5	100	100	100	100	100	100	100
**Georgia_2007_1**	100	100	100	100	99.63	100	100	99.5	100	100	100	100	100	100	100
**Estonia_2014**	100	40.62	100	100	100	100	100	99.5	100	100	100	100	100	100	100
**CN_2019_InnerMongolia-AES01**	100	100	100	97.26	100	100	100	99.5	100	100	99.49	99.19	100	100	100
**China_AnhuiXCGQ**	100	100	100	100	100	100	100	99.5	100	100	100	100	100	100	100
**Benin_97_1**	96.34	98.31	100	98.59	97.39	90.16	51.38	98.5	98.1	99.91	97.95	95.97	98.82	100	100
**Belgium_Etalle_wb_2018**	100	100	100	100	100	100	100	99.5	100	100	100	100	100	100	100
**ASFV-wBS01**	98.94	100	100	100	100	99.45	99.37	99.5	99.52	99.91	100	100	100	100	100
**ASFV_Wuhan_2019-1**	100	100	100	100	100	100	98.76	99.5	100	100	100	100	100	99.86	100
**ASFV_pig_China_CAS19-01_2019**	100	100	100	100	100	100	100	99.5	100	100	100	100	100	100	100
**ASFV_LT14_1490**	100	100	100	100	99.63	100	100	99	100	100	100	100	100	100	100
**ASFV_HU_2018**	100	100	100	100	100	100	100	99.5	100	100	100	100	100	100	99.68
